# Visuomotor Adaptation of Lower Extremity Movements During Virtual Ball-Kicking Task

**DOI:** 10.3389/fspor.2022.883656

**Published:** 2022-06-23

**Authors:** Mai Moriyama, Motoki Kouzaki, Shota Hagio

**Affiliations:** ^1^Laboratory of Neurophysiology, Graduate School of Human and Environmental Studies, Kyoto University, Kyoto, Japan; ^2^Unit of Synergetic Studies for Space, Kyoto University, Kyoto, Japan

**Keywords:** lower limb movement, motor learning, visuomotor rotation, goal-directed movement, virtual reality

## Abstract

Sophisticated soccer players can skillfully manipulate a ball with their feet depending on the external environment. This ability of goal-directed control in the lower limbs has not been fully elucidated, although upper limb movements have been studied extensively using motor adaptation tasks. The purpose of this study was to clarify how the goal-directed movements of the lower limbs is acquired by conducting an experiment of visuomotor adaptation in ball-kicking movements. In this study, healthy young participants with and without experience playing soccer or futsal performed ball-kicking movements. They were instructed to move a cursor representing the right foot position and shoot a virtual ball to a target on a display in front of them. During the learning trials, the trajectories of the virtual ball were rotated by 15° either clockwise or counterclockwise relative to the actual ball direction. As a result, participants adapted their lower limb movements to novel visuomotor perturbation regardless of the soccer playing experience, and changed their whole trajectories not just the kicking position during adaptation. These results indicate that the goal-directed lower limb movements can be adapted to the novel environment. Moreover, it was suggested that fundamental structure of visuomotor adaptation is common between goal-directed movements in the upper and lower limbs.

## Introduction

Sophisticated football players can run across the playing field while skillfully manipulating the ball with their feet. Lower extremities play a primary role in controlling upright standing or locomotion that stabilizes or translates a body's center of mass (CoM) (Winter, 1995; Runge et al., 1999). In addition to the function involved with the CoM, the endpoint movements of the lower extremities can also be controlled toward a desired target, such as kicking a ball while playing soccer. Previous studies have demonstrated functional force control in the lower extremities to the desired multidirectional targets (Jacobs and Van Ingen Schenau, 1992; Hagio and Kouzaki, 2014). However, how the sensorimotor system learns goal-directed movements in the lower extremities is not fully understood.

Sensorimotor adaptation in the lower extremities has been studied with the split-belt adaptation task, where participants walk on belts moving at different speeds under each foot (Roemmich et al., 2016; Day et al., 2018). It was shown that lower-limb movements can be flexibly adapted to the novel environment. However, these studies investigated the adaptation of locomotion, not goal-directed movements in the lower extremity. Sensorimotor adaptation of goal-directed movements has been investigated using reaching tasks with the introduction of visuomotor or force-field perturbations (Shadmehr and Mussa-ivaldi, 1994; Krakauer et al., 2000). Since these tasks are upper extremity motor tasks, it is still unclear whether the process of adaptation in the goal-directed movements is common between the upper and lower extremity.

Goal-directed movements need appropriate sensorimotor transformation, i.e., transforming sensory input to the desired motor output, which is updated based on the experience of errors between the desired and actual movement (Wolpert et al., 1995; Krakauer et al., 1999; Wolpert and Ghahramani, 2000; Lalazar and Vaadia, 2008). Considering the differences in the characteristics of the upper and lower extremities, such as the moment of inertia of the segments (Winter, 2009) and the sensorimotor delay (Kandel et al., 2021), it is probable that the properties of the sensorimotor transformation are different between the upper and lower limb movements. Therefore, it is necessary to investigate the sensorimotor learning in the lower limb apart from the upper-limb motor learning.

The purpose in this study was to clarify how we adapt goal-directed movements in the lower limb. Here, we constructed a virtual ball-kicking task where the trajectory of the kicked ball was rotated relative to the actual trajectory. Thereby, we investigated the process of adaptation to the novel visuomotor environment in the lower limb control.

## Materials and Methods

### Participants

Sixteen adults (10 males and 6 females) participated in this experiment. Half of the participants were “experts,” who had played soccer or futsal for at least 3 years, and the others were untrained “novices.” We recruited the experts and novices in order to clarify whether learning visuomotor perturbation with the lower limb requires familiarity with the lower limb control. Experts [6 males and 2 females, age = 22.6 ± 0.92 years, height = 169.0 ± 10.8 cm, weight = 62.8 ± 8.2 kg, mean ± standard deviation (SD)] had played soccer or futsal for at least 3 years (6.75 ± 2.82 years) in club activities at the college, high school, or junior high school level. The remaining 8 participants (4 males and 4 females, age = 22.5 ± 1.07 years, height = 170.0 ± 8.0 cm, weight = 64.0 ± 11.6 kg) were defined as novices who had never experienced both soccer and futsal. All participants were right-footed and manipulated their dominant leg. They gave informed consent, and the experimental procedures were in accordance with the Declaration of Helsinki and approved by the Ethics Committee for Human Experimentation at the Graduate School of Human and Environmental Studies, Kyoto University (19-H-25).

### Experimental Setup

**Figure 1:**
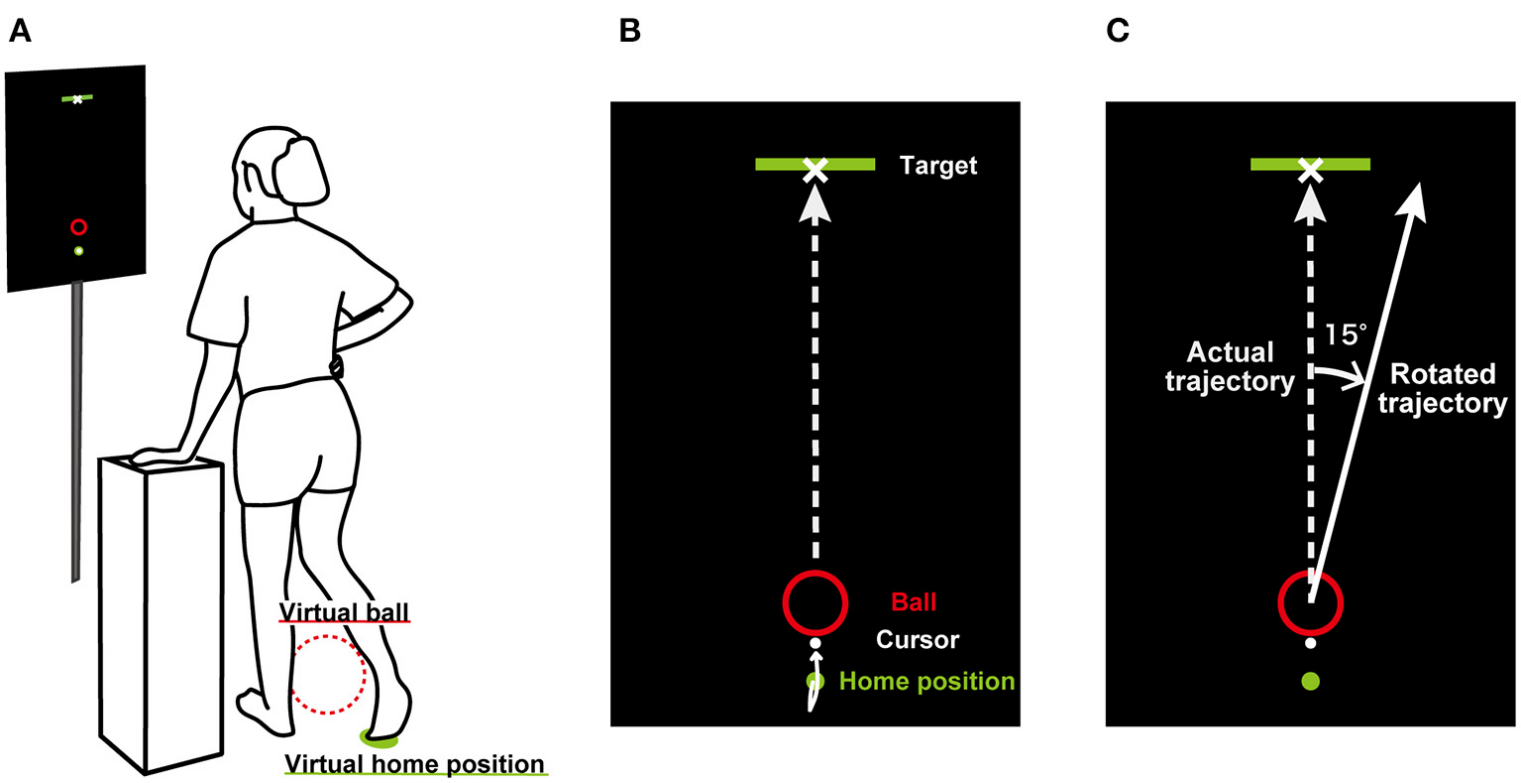
Experimental setup. **(A)** shows how participants performed a ball-kicking task. They swung their right leg while standing with their right hand on their hip and their left hand comfortably placed on a table. They were asked to swing their right foot and kick a virtual ball in the display so that the virtual ball could hit the target. **(B)** illustrates the display shown in front of the participants. On the screen, the locations of the target, virtual ball, home position, and cursor on the horizontal plane were displayed. The virtual ball moved in constant velocity linear motion depending on the kicking position and the right foot velocity. **(C)** shows the rotated trajectory during the perturbation period.

Participants stood on their left leg with their right hand on their hip and their left hand comfortably placed on a table (Figure 1A). The height of the table was adjusted depending on each participant. A 27-inch LCD monitor (60 Hz) was placed 1.5 m in front of the participants at eye level and displayed a target (10 cm × 50 cm), virtual ball (1 cm radius red circle), home position (0.5 cm radius green circle), and cursor (0.5 cm radius white circle) (Figure 1B). The target was displayed above the screen, the ball was 40 cm below the target, and the home position was 4 cm below the ball. The distance between the home position and the center of the virtual ball in reality was 50 cm. The horizontal right foot position was captured by the real-time motion tracking system used in our previous study (Hagio and Kouzaki, 2020). Trajectories of a rigid body created by three infrared reflective markers attached on the instep of the right foot were sampled at 100 Hz using the three-dimensional optical motion capture system (OptiTrack V100, Natural Point Inc., Corvallis, United States) with 12 cameras. The rigid body coordinates were streamed from Motive 2.0.2 software (Natural Point Inc., Oregon, United States) to MATLAB (R2019a, The MathWorks Inc., Natick, MA, United States) in real time and displayed in the 2-dimensional coordinates of a MATLAB figure as a white cursor (0.5 cm radius).

### Virtual Ball-Kicking Task

Participants moved the cursor to hit the virtual ball displayed in the monitor toward the center of the forward target. The experimental task was assumed to be soccer ball kicking. They were instructed to move the cursor backward at first before hitting the virtual ball and then move toward the virtual ball. The trajectories of the virtual ball were determined depending on the cursor position and the velocity was immediately measured when the cursor hit the virtual ball. After the hitting, the virtual ball position, (*x, y*), was translated following constant velocity linear motion:


$$\left[\begin{array}{c} \;x\;\\ y \end{array}\right]=\;K\frac{m_{\;leg}}{m_{\;ball}}\left[\begin{array}{c} \;V_{x}\;\\ V_{y} \end{array}\right]t\;$$

where *V*_*x*_ and *V*_*y*_ are the *x*- and *y*-components of the foot velocity transformed to the direction of the center of the ball, and *m*_*leg*_ and *m*_*ball*_ indicate the mass of the leg and the ball, respectively. *t* represents the time elapsed after the hitting. The body mass and mass of the leg were regarded as 50 kg and 16.1% of the body weight (Winter, 2009), respectively; *m*_*leg*_ was set as 8.05 kg. *m*_*ball*_ was determined to be 0.45 kg, corresponding to the weight of the official soccer ball. A constant parameter, *K*, was defined as 0.45 so that the ball moves naturally on the screen. Although the ball movement following constant velocity linear motion and no contact information between the ball and foot were different from the actual ball-kicking situation, the simple visuomotor task was intended to investigate the visuomotor control of the goal-directed lower limb movements.

### Experimental Procedure

Before each trial, participants moved the cursor representing their right foot position to the home position on the monitor. After their right foot was held in the start position (within 2.5 cm in reality), a green rectangular target appeared. After 0.5–1.5 s, the target's color turned from green to magenta, and the home position disappeared, which was the cue that participants should initiate the movement to kick the virtual ball. After each trial, the velocity of the right foot after kicking the virtual ball was immediately shown on the screen. The participants were instructed to control the foot velocity to between 2.5 and 3.5 m/s.

This experiment was conducted for 2 days. The main experiment was performed on the second day, and these trials were used for analysis. On the first day, participants practiced the ball-kicking task 4–7 sets (40 trials per set) to get familiar with the task. There was a rest for 90 s after each set. After 160 trials, the practice was terminated when participants were able to hit the target and reduce the variability of ball direction, and the experimenter judged that the bias of ball direction had been stable during a set. The number of practice trials was decided by the same experimenter. On the second day, there were 400 trials (40 trials × 10 sets), and they were divided into three phases: baseline, perturbation, and washout periods. These phases consisted of 130, 160, and 110 trials, respectively. The length of each phase was determined based on pilot data so that the participants could adapt to the novel visuomotor environment; after-effects appeared after the perturbation period.

In the perturbation period, visuomotor perturbation was introduced without telling participants. Visuomotor perturbations have been used in reaching tasks, in which the cursor corresponding to the location of the subject's hand is rotated (Krakauer et al., 1999; McDougle et al., 2015). In the current study, the trajectories of the ball were rotated by 15° either clockwise or counterclockwise relative to the actual trajectories as follows (Figure 1C):


$$\left[\begin{array}{c} \;x^{\prime }\;\\ y^{\prime } \end{array}\right]=\;\left[\begin{array}{cc} cos\theta & sin\theta \\ -sin\theta & cos\theta \end{array}\right]\left[\begin{array}{c} \;x\;\\ y \end{array}\right]\;$$

where *x', y'* corresponds to the rotated position of the ball; *x, y* corresponds to the actual position of the ball; and θ denotes the perturbation angle about the center of the ball at the start position equal to either 15° or −15°. The direction of visuomotor perturbation was counterbalanced across participants. All the experimental tasks were implemented using a custom-made MATLAB (R2019a, The MathWorks Inc., Natick, MA, United States) script.

### Data Analysis for the Virtual Ball

The error on a given trial was calculated as the angular difference between a line from the starting position of the virtual ball to the target and a line in which the virtual ball moved. The mean error in the last 50 baseline trials was subtracted from the errors of all trials for each participant. In the case of the clockwise perturbation group, the errors were multiplied by −1 so that the positive error corresponded to the deviation of the virtual ball in the direction of the perturbation and vice versa. We quantified the mean error of the first 10 trials of the washout period as an aftereffect.

### Outlier Analysis

Individual trials were excluded as outliers if the velocity of the right foot was smaller than 1.5 m/s or larger than 5.5 m/s. After that, the error thresholds of each participant at each phase (baseline, perturbation, and washout periods) of the task were determined as follows: in the baseline period, thresholds were defined as the mean ± 3 SD of errors during the last 50 baseline trials (= λ), while in the perturbation and washout periods, the predicted error (15°) was added to one of the thresholds (λ) of the side in which the effect of perturbation would appear [i.e., the direction where the error increased just after the perturbation was turned on (or off)]. If the error exceeded these thresholds, the data were removed. This resulted in the omission of <1.44% of trials on Day 2 (92 trials out of 6,400 trials).

### Analysis of the Right Foot Trajectories

We focused on the trajectories of the cursor representing the right foot marker on the horizontal plane. The time series of the cursor position was filtered using a zero-lag, fourth-order Butterworth filter with a low-pass filter cutoff of 8 Hz. The onset of the data was defined as the moment when the movement exceeded 10% of peak velocity after leaving the starting position. The movement offset was immediately measured when the cursor contacted the virtual ball.

### Statistics

All statistical tests were executed using functions in the Statistics Toolbox of MATLAB (R2019a). We performed a one-sample *t*-test for the size of the initial error in the washout period for each group (novices and experts). The difference was assumed to be statistically significant at an α threshold of 0.05.

## Results

**Figure 2:**
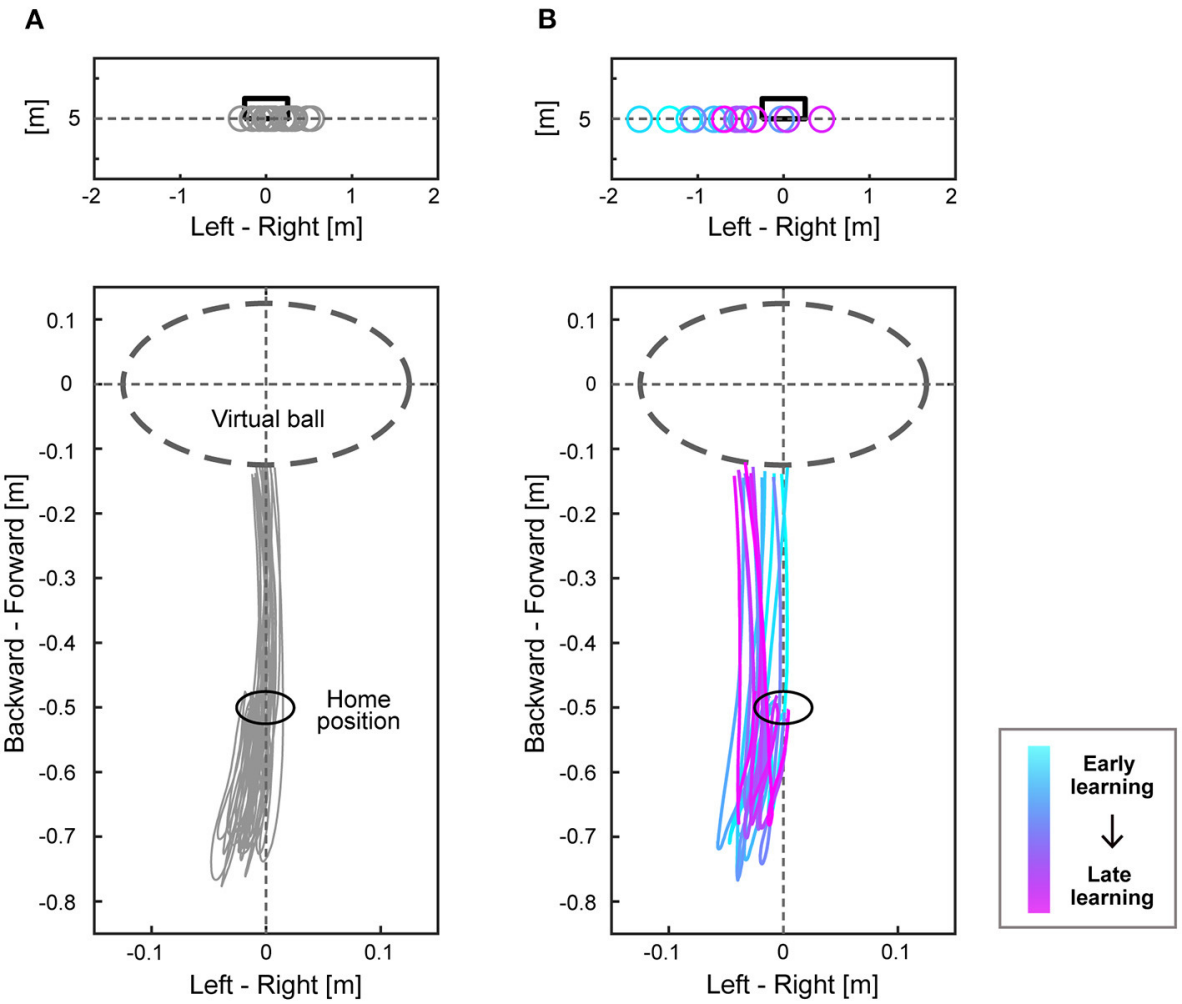
Endpoints of the virtual ball and trajectories of the right foot. The upper figures of **(A,B)** show the endpoints of the virtual ball of a representative expert subject of the counterclockwise group. The black box represents the target, and each circle shows endpoints of the virtual ball at each trial. The lower figures of **(A,B)** show trajectories of the right foot on the horizontal plane when the participant swung their right foot backward from the home position and kicked the virtual ball. The dashed line circle represents the location of a virtual ball. **(A)** shows the results during the last 20 trials of the baseline period, and **(B)** shows each result of every 10 trials during the perturbation period (16 trajectories). The color changes from cyan to magenta as the trial proceeds.

Participants practiced the ball-kicking task on the first day. The mean absolute errors of the virtual ball and the variability of errors decreased through practice (Supplementary Table 1). On the second day, participants performed the main task consisting of 400 trials. The endpoint of the virtual ball and right-foot trajectories in the last 20 trials of the baseline period are shown for a representative expert (Figure 2A). The virtual ball position varied around the target, and the participant repeated a similar trajectory (Figure 2A). The virtual ball position and foot trajectories are also shown in 16 trials (every 10 trials) during the perturbation period when visuomotor rotation to a counterclockwise direction was applied for a representative expert (Figure 2B). After the perturbation was introduced, the virtual ball position deviated in the direction corresponding to the rotational perturbation and then gradually approached the target, which was accompanied by a change in the foot trajectories (Figure 2B).

### Errors of the Virtual Ball

**Figure 3:**
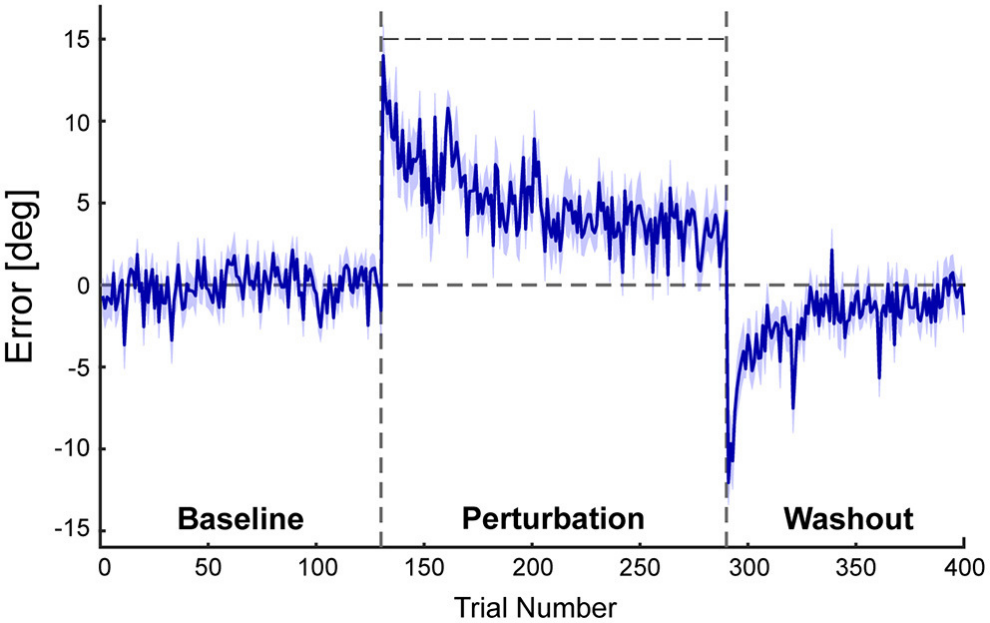
Results of the errors between the center of the target and the endpoint of the virtual ball. This figure shows angular errors between the center of the target and the endpoint of the virtual ball. A positive error corresponds to the deviation of the virtual ball in the direction of the perturbation and vice versa. Blue lines represent the mean errors of all participants. The shaded area represents the standard error of the mean (SEM). Four hundred trials consisted of three phases: the baseline period (130 trials), perturbation period (160 trials), and washout period (110 trials). A horizontal line during the perturbation period (trial 131–290) indicates the perturbation of 15°.

Figure 3 shows the mean errors in ball direction. Each individual participant's learning curve is shown in Supplementary Figure 1. Once the perturbation was introduced after the baseline period, the errors increased in the direction corresponding to the perturbation and then decreased by repeating the trials. Participants required about 100–120 trials to reduce the errors of the virtual ball. After the perturbation was turned off, errors were observed in the negative direction and then modified to the level equivalent to the baseline period. The size of the initial error in the washout period was significantly different from zero (novices, −7.07 ± 3.66°; *p* < 0.001; experts, −6.84 ± 2.75°; *p* < 0.001). This after effect indicates that participants adapted their lower limb movements to the novel visuomotor environment.

### Foot Trajectories

**Figure 4:**
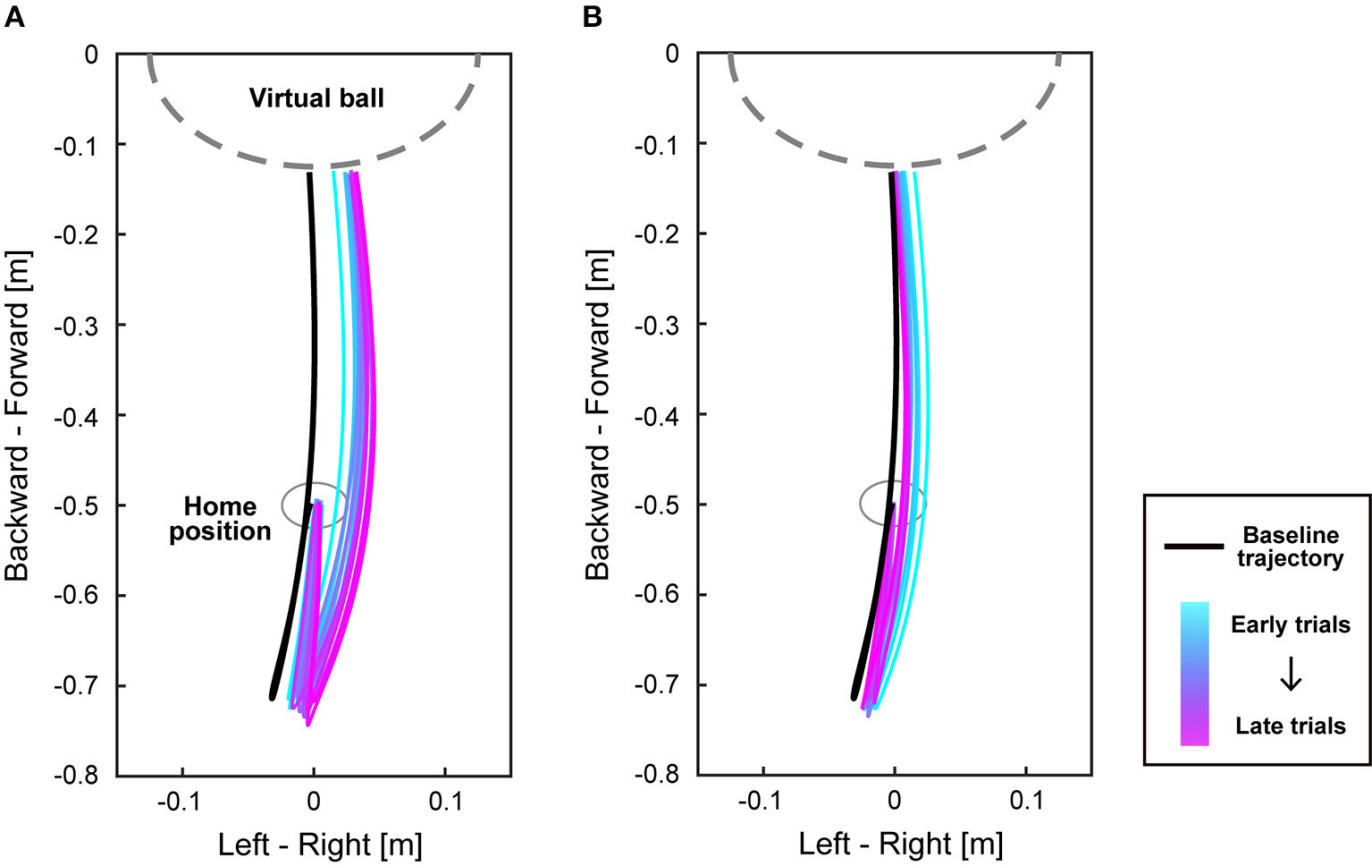
Trajectories of the right foot during adaptation. **(A,B)** show the mean trajectories of the right foot on the horizontal plane from when participants started to move from the home position until their foot contacted the virtual ball. The black line represents the baseline trajectory of each group, and the colored lines show the mean trajectories of every 10 trials during the perturbation period **(A)** and the washout period **(B)** in the clockwise perturbation group. As the trials proceed, the color of the trajectory changes from cyan to magenta. The gray dashed line represents the location of a virtual ball.

We next investigated the movement trajectories of the right foot. Each participant's trajectories of the right foot during the perturbation period are shown in Supplementary Figure 2. Figure 4 shows the mean trajectories of the clockwise perturbation group during the perturbation and the washout period. During adaptation (Figure 4A), trajectories of the right foot were gradually changed from the baseline trajectory (a black line) to the direction corresponding to the perturbation so that participants could compensate for the increased error of the virtual ball position. Particularly, the foot movements were changed not only at the end of the trajectories, i.e., near the virtual ball, but for the entire trajectories both in backward and forward directions. After the perturbation was turned off, the trajectories gradually approached the baseline trajectory (Figure 4B).

## Discussion

In this study, we aimed to investigate how the goal-directed control of the lower limb is adapted. The results demonstrated that both the novices and experts can adapt their lower limb movements to novel visuomotor perturbation (Figure 3). Furthermore, the foot trajectories during adaptation showed that not only the trajectories near the kicking position but the whole trajectories were changed (Figure 4).

Participants in the current study were not told about the existence of the perturbation and told to hit the target with the virtual ball throughout the experiment. However, errors at the beginning of the washout period were significantly different from 0 (Figure 3). Moreover, participants couldn't immediately change their trajectories to the baseline trajectory after the perturbation was turned off (Figure 4). These results indicate that the current task elicited implicit adaptation. While, it should be noted that the washout trials may be contaminated with some explicit strategy. Participants in the present study were not restricted from aiming in other directions rather than toward the target (re-aiming strategy) so that the virtual ball could hit the target during the washout period. To isolate implicit adaptation, future studies should introduce other paradigms such as restricting participants from using explicit strategy or providing clamped visual feedback which is pre-determined and irrelevant to the task (Morehead et al., 2017; Tsay et al., 2020).

The visuomotor rotation in the current study presented a cursor representing veridical (not rotated) foot position and a rotated trajectory of the virtual ball. Accordingly, there was no sensory prediction error, a discrepancy between predicted and actual feedback of the effector, but a task error, which is a difference between actual performance and the task goal. This implies that implicit adaptation is induced by a discrepancy between predicted and actual feedback of the motor performance (predicted and perturbated trajectories of the virtual ball) rather than sensory prediction error (Ranjan and Smith, 2020). While, implicit adaptation has been thought to be elicited by sensory prediction error (Mazzoni and Krakauer, 2006; Shadmehr et al., 2010). Additionally, it has been shown that a visuomotor rotation task without sensory prediction error but with only task error does not induce trial-by-trial implicit adaptation (Tsay et al., 2022). Further studies are required to specifically investigate implicit adaptation without sensory prediction error.

During the perturbation period, the shape of the foot trajectory was changed from the baseline trajectory (Figure 4). Previous studies showed that the postadaptation trajectory in the visuomotor rotation task of the upper limb was the rotated trajectory of the baseline trajectory, resulting in a consistent shape before and after adaptation (Novick and Vaadia, 2011; Hirashima and Nozaki, 2012). It was also demonstrated that the directional tuning of muscle activity is rotated depending on the visuomotor perturbation (Gentner et al., 2013; De Marchis et al., 2018). However, such rotation did not appear to be applied to the visuomotor adaptation in the lower limb. Instead, the whole trajectories seemed to be more curved toward the direction of the perturbation (Figure 4). This might be due to the difference in the biomechanical properties or the degrees of freedom between the upper and lower limbs.

To our knowledge, this is the first study on visuomotor adaptation of goal-directed movement in the lower limb, although visuomotor adaptation of goal-directed movement in the upper limb has been studied for decades (Shadmehr and Mussa-ivaldi, 1994; Krakauer et al., 2000). There are many differences between the upper and lower limbs in motor control, such as the length of sensorimotor delay (Kandel et al., 2021) and the size of the moment of inertia of the segments (Winter, 2009), and moreover, standing posture must be maintained during lower-limb tasks. Indeed, such differences might be related to the fact that while the postadaptation trajectory of the visuomotor rotation task with the upper limb was rotated (Novick and Vaadia, 2011; Hirashima and Nozaki, 2012), the structure of the postadaptation trajectory in the lower limb was changed from the baseline trajectory rather than a simple rotation (Figure 4). Despite such dissimilarities, the time course of errors (Figure 3) was similar to previous studies on visuomotor learning of reaching movements (Mazzoni and Krakauer, 2006; Taylor et al., 2014; Singh et al., 2016), suggesting that the fundamental structure of visuomotor adaptation is common between goal-directed movements in the lower and upper limbs.

In the current study, we recruited the novices and experts to clarify whether learning visuomotor perturbation with the lower limb requires familiarity with the lower limb control. As a result, both novices and experts could adapt to the visuomotor rotation (Figure 3). Previous studies on motor learning in the upper limb movements have demonstrated that sensorimotor experience of task-related movements had an effect on motor learning of visuomotor rotation (Leukel et al., 2015; Kast and Leukel, 2016; Hewitson et al., 2020). It has been shown that experts rely on explicit strategies more than novices during adaptation (Leukel et al., 2015; Hewitson et al., 2020). Hence, it is possible that there was some difference in the contribution of explicit and implicit processes during the lower-limb adaptation between the novices and experts, which should be examined in future studies.

In conclusion, it was demonstrated that lower-limb movement can be adapted to the novel visuomotor environment. This finding suggests that the fundamental structure of visuomotor adaptation in goal-directed movements is universal regardless of the effector.

## Data Availability Statement

The raw data supporting the conclusions of this article will be made available by the authors, without undue reservation.

## Ethics Statement

The studies involving human participants were reviewed and approved by the Ethics Committee for Human Experimentation at the Graduate School of Human and Environmental Studies, Kyoto University. The patients/participants provided their written informed consent to participate in this study. Written informed consent was obtained from the individual(s) for the publication of any potentially identifiable images or data included in this article.

## Author Contributions

MM, MK, and SH conceived the research, interpreted the data, revised the manuscript, and approved the final version to be published. MM and SH designed the experiment and collected and analyzed the data. MM prepared figures and drafted manuscript. SH edited the manuscript. All authors contributed to the article and approved the submitted version.

## Funding

This work was supported by Japan Society for the Promotion of Science (JSPS), Grant-in Aid for Scientific Research (B) (18H03165, MK).

## Conflict of Interest

The authors declare that the research was conducted in the absence of any commercial or financial relationships that could be construed as a potential conflict of interest.

## Publisher's Note

All claims expressed in this article are solely those of the authors and do not necessarily represent those of their affiliated organizations, or those of the publisher, the editors and the reviewers. Any product that may be evaluated in this article, or claim that may be made by its manufacturer, is not guaranteed or endorsed by the publisher.
