# Supplementary material for: Visuomotor Adaptation of Lower Extremity Movements During Virtual Ball-Kicking Task
Source: Front Sports Act Living. 2022 Jun 23;4:883656. doi: 10.3389/fspor.2022.883656 (PMC9259925; doi:10.3389/fspor.2022.883656)
Supplement: Supplementary file 1 [file Image_1.pdf]

Supplementary Material

1 Supplementary Figures

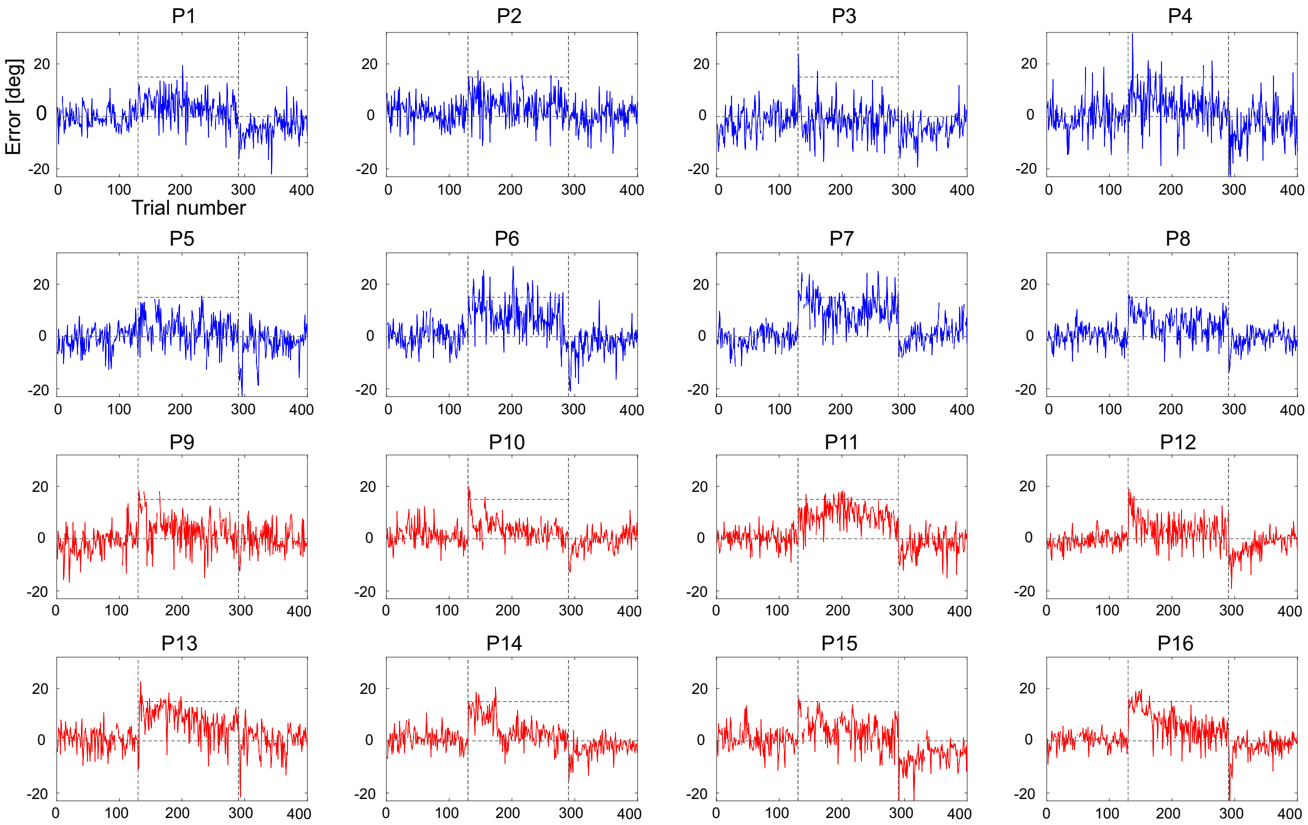

Supplementary Figure 1. Results of the errors between the center of the target and the endpoint of the virtual ball

This figure shows angular errors between the center of the target and the endpoint of the virtual ball for each participant. A positive error corresponds to the deviation of the virtual ball in the direction of the perturbation and vice versa. Upper graphs indicate novices (P1-P8; blue), and lower graphs indicate experts (P9-P16; red). A horizontal line during the perturbation period (trial 131-290) indicates the perturbation of 15°.

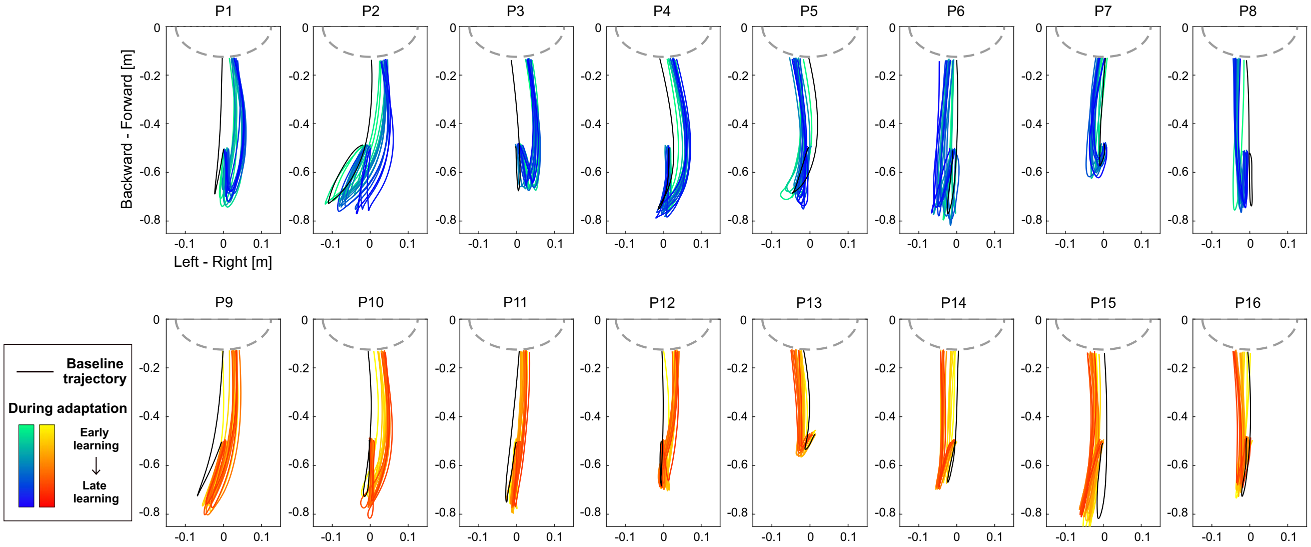

Supplementary Figure 2. Trajectories of the right foot during adaptation

This figure shows the mean trajectories of the right foot on the horizontal plane for each participant from when participants started to move from the home position until their foot contacted the virtual ball. The black line represents the baseline trajectory of each participant, and the colored lines show the mean trajectories of every 10 trials during the perturbation period. The left half of the participants (P1-P4, P9-P12) were the clockwise-perturbation group, and the right half of the participants (P5-P8, P13-P16) were the counterclockwise-perturbation group. As the trials proceed, the color of trajectories of the novices changes from green to blue (P1-P8) and that of the experts changes from yellow to red (P9-P16). The gray dashed line represents the location of a virtual ball.
