# Supplementary material for: Visuomotor Adaptation of Lower Extremity Movements During Virtual Ball-Kicking Task
Source: Front Sports Act Living. 2022 Jun 23;4:883656. doi: 10.3389/fspor.2022.883656 (PMC9259925; doi:10.3389/fspor.2022.883656)
Supplement: Supplementary file 2 [file Table_1.docx]

Supplementary Material

|  |  | Mean of Absolute Errors [deg] | Standard Deviation of Errors [deg] |
| --- | --- | --- | --- |
| Novices | Initial 50 trials | 4.23 $\pm$ 1.03 | 4.80 $\pm$1.37 |
|  | Last 50 trials | 3.89 $\pm$1.15 | 4.57 $\pm$1.03 |
| Experts | Initial 50 trials | 4.00 $\pm$1.15 | 4.46 $\pm$1.26 |
|  | Last 50 trials | 3.21 $\pm$ 0.92 | 3.78 $\pm$1.15 |

# Supplementary Table 1. Results of the errors on Day 1

The table shows the results of the angular errors between the center of the target and the endpoint of the virtual ball during the practice trial on Day 1. The mean of the absolute errors and the standard deviation of errors in the initial 50 trials and the last 50 trials were averaged for the novices and experts.
